# Differential Activity of Stanniocalcin in Male and Female Fresh Water Teleost *Mastacembelus armatus* (Lacepede) during Gonadal Maturation

**DOI:** 10.1371/journal.pone.0101439

**Published:** 2014-07-08

**Authors:** Sushant Kumar Verma, Abdul Alim

**Affiliations:** Co-operative College, Ranchi University, Ranchi, Jharkhand, India; CNRS, France

## Abstract

The present study was carried out to analyze the differences in the activity of hormone stanniocalcin (STC) between male and female fishes of *Mastacembelus armatus* during their gonadal cycle. A large variation in nuclear diameter of cells of corpuscles of Stannius (CS) were recorded in relation to testicular cycle as well as ovarian cycle which indicates that the cellular activity varied with different phases of reproductive cycle in both male and female fish. Similar changes in nuclear diameter of CS cells were also observed after 17alpha-methyltestosterone administration in males and 17 β-estradiol administrations in females. A positive correlation was observed between plasma STC levels, gonadosomatic index (GSI) and the sex steroids in both sexes, suggesting that STC has a role in the processes involved in gonadal development. In addition females showed remarkable changes in plasma calcium level during gonadal cycle while no such change for males were observed. In females the plasma calcium level estimated during different phases of reproductive cycle indicates positive correlation between plasma level of calcium and gonad growth. Thus hyperactivity of CS cells was noted in both male and female fishes during gonadal cycle along with the differences in the activity of STC as well. In female it may act as hypocalcemic factor and bring the level of calcium to normal which increases during preparatory and pre spawning phases to fulfill the increased demand of calcium for vitellogenesis. However data of male fishes indicated that plasma STC concentration varied widely during gonadal cycle but showed no consistent relationship to plasma calcium level.

## Introduction

The corpuscles of Stannius (CS) are unique calcitropic endocrine gland found associated with kidney of teleostean and holostean fishes. It synthesizes and secretes hormone stanniocalcin-1 (STC-1) and stanniocalcin-2 (STC-2). Stanniectomy in eels results in significant hypercalcemia [1], while on the other hand administration of CS extracts in to stanniectomized eels restored the serum electrolyte level to normal [2]. It has been observed that auto transplantation of CS in eels brings the plasma calcium level to normal [3]. STC-1 maintain calcium homeostasis by inhibiting calcium transport through gills [4], reducing calcium uptake through intestine [5], and stimulating phosphate reabsorption by renal proximal tubules [6]. Synthesis and secretion of STC-1 by the CS have been shown to be sensitive to extracellular ionized calcium concentration [7]. It is now a well established fact that calcium homeostasis in fishes is mainly mediated through the secretions of CS [8]–[12]. A second stanniocalcin (STC-2) has been identified in fishes [13], however no report exists in evidence of its role in calcium regulation [14].

Plasma calcium rises during gonadal maturation in fishes [15]. There exists a difference in the increase of plasma calcium level with respect to sex of the fish. It is more pronounced in females as reported from time to time by various workers [16]–[18]. The increase in plasma calcium during ovarian maturation is due to increased secretion of estrogen from the ovary [19], [20] as administration of estradiol was found to induce hypercalcemia [15], [20]. Estradiol increases the level of plasma calcium by acting directly on gills [21] as well as on intestine [22]. It may act indirectly via some endocrine factor like PTHrP or a related factor responsive to E2 [23]. Most of the studies indicated that there exists no correlation between serum calcium level and testicular maturation in fishes [24]–[26]. However Woodhead and Woodhead [25] suggested a positive correlation between blood calcium level and testicular maturation in sea cod.

CS becomes activated during gonadal maturation and this response is more pronounced in case of females [27]. Increase in the activity of CS in male is not common but has been reported earlier by Balbontin et al. [28]. This hyperactivity of CS during gonadal maturation may due to elevated calcium level [29] or it may stimulate gonadal development as reported by Hiroi [30].

As *Mastacembelus armatus* is one among the economically important species in rural parts of India [31] it becomes necessary to know every aspect of its reproductive physiology. The role of STC in females *M. armatus* has been established as calcium regulating factor by us in our earlier work [32], however no such report exists in this regard evaluating the gender specific differential activity of STC during gonadal maturation in *M. armatus*. Therefore the present study was undertaken to analyze the differential activity of STC in male and female fresh water teleost *M. armatus* (Lacepede) during gonadal maturation. We hypothesized that positive correlation between plasma calcium and STC level during gonadal maturation will indicate towards its definite role in calcium homeostasis while on the other hand variation in plasma STC level during different phases of gonadal cycle without any significant change in the level of calcium will indicate towards its possible role in gonadal maturation.

## Materials and Methods

### Fish Collection and Maintenance

Ten healthy and adult specimens of fish *M. armatus* were collected every month throughout the year with the help of fishermen. The live fishes were brought to the laboratory and were acclimatized to laboratory conditions for 15 days in plastic pool tanks having size of 90 cm diameter and 60 cms height. During this period, fish were fed with live earthworms and boiled eggs. Water was replaced every 24 h to remove fecal matter, other waste materials and residue food particles as well as to maintain suitable environment for fishes with sufficient oxygen. The tank water was maintained with following composition: temperature = 28°C±1.0°C, salinity = 0.6±0.02 ppt, total hardness = 28±0.05 mg/l, pH = 6.5±0.2 units, dissolved oxygen = 6.6±0.01 mg/l. The photoperiod maintained through the entire experiment was 12:12 h. The average (±SD) body weight and length of male and female fishes were 300±2.50 g, 26±0.35 cm and 350±2.86 g, 31±0.35 cm respectively. After anesthetized with phenoxyethanol the tail was severed and the blood samples were collected from the caudal vessels using a heparinized syringe for estimation of plasma calcium, 17 β-estradiol, testosterone and STC level. The fishes were sacrificed, gonads were taken out after which they were weighed (gm) for the determination of gonadosomatic index and fixed in Bouin's solution for 12 to 16 hours (depending upon the size of the tissue) after which they were completely dehydrated. Kidney along with CS was also taken out and fixed in Bouin's solution for 24 hours. Paraffin blocks of both gonads and kidney were prepared and sections of 5–7 µm were made using a microtome. Maturity stages of gonads were determined by studying histological changes after staining sections in haematoxylin and counterstaining in eosin. The sections of kidney along with CS were also stained in haematoxylin and counterstained in eosin.

Nuclear diameter of cells of CS (µm) was measured by image analyzer microscope (Metavis image analyzing system with Meltmage Lx Software). 50 nuclei were randomly selected from every fifth section of the gland. Total number of the nuclei measured was always more than 300 for each individual.

### Plasma Calcium Estimation

After centrifugation in cooling centrifuge (maintained at 4°C, 4000 rpm for 5 minutes) plasma was analyzed for total plasma calcium concentrations using calcium kit (Sigma Diagnostics).

### Enzyme Linked Immunosorbent Assay

A competitive ELISA technique [33] which is based on competition between free STC in standard or plasma samples and STC immobilized on microtiter plates for the STC antibodies was used for determination of plasma STC level.

### Radioimmunoassay

Plasma testosterone and 17 β-estradiol levels were determined by RIA method [34]–[35] following Guerriero et al. [36]. The sensitivity of testosterone was 7 pg (intraassay, 6%; interassay, 12%), and that of 17 β-estradiol was 5 pg (intra-assay, 8%; interassay, 12%). The antibody used for testosterone determinations cross-reacted with dihydrotestosterone, and therefore the data are reported as androgens [37].

### Experiment

To determine the differential activity of CS cells during gonadal cycle, an experimental set up was designed. 24 live, (12 male and 12 female) adult and healthy specimens of *M. armatus* were collected from local fishermen, during the month of December which is the resting phase for gonads. Fish were acclimatized to laboratory conditions for 15 days in plastic pool tanks having size of 90 cm diameter and 60 cms height. During this period, fish were fed with live earthworms and boiled eggs. Water was replaced every 24 h to remove fecal matter, other waste materials and residue food pellets as well as to maintain suitable environment for fishes with sufficient oxygen. The aquarium water was maintained with following composition: temperature = 28°C±1.0°C, salinity = 0.6±0.02 ppt, total hardness = 28±0.05 mg/l, pH = 6.5±0.2 units, dissolved oxygen = 6.6±0.01 mg/l. The photoperiod maintained through the entire experiment was 12:12 h. After 15 days the fishes were divided in four groups, two groups consisting of 06 female each and other two groups with 06 males each and kept in four separate aquaria of 100 L capacity. Out of the two groups of females one group was injected with 0.1 ml of vehicle (peanut oil), the other group was administrated with 100 µg of 17 β-estradiol (sigma) in 0.1 ml of vehicle. One group of males was injected with 0.1 ml of vehicle (peanut oil), while other group of males was injected with 100 µg of 17alpha-methyltestosterone (sigma) in 0.1 ml of vehicle. The fishes were injected intraperitonially on alternate days and injections were given at the same time of the day to avoid diurnal variations. The blood samples were analyzed for plasma calcium, 17 β-estradiol, testosterone and STC level estimation after 15 days. At the same time the CS was also removed for histological analysis.

### Statistical Analysis

Distribution parameters are presented as means and Standard Deviation (SD). Assignment of data correlation was done by Pearson tests, and the relationships between total plasma calcium and STC were evaluated by linear regression. The accepted statistical significance level was P<0.05.

### Ethics Statement

The study plan was approved by the ethical committee of Ranchi University, Ranchi (Jharkhand). Experiments were conducted according to guidelines approved by the relevant authorities in India (Indian National Science Academy and Indian Council of Medical Research).

## Results

### Gonadal Cycle

On the basis of morphological and histological changes occurring in gonads five different stages for gonadal cycle of *M. armatus* have been identified- Phase 1 or Resting phase (December–February), Phase II or Preparatory Phase (March–May), Phase III or Pre spawning phase (June–early July), Phase IV or Spawning phase (Late July- September) and Phase V or Post spawning phase (October–November). Morphological and histological characteristics of gonads during these phases are shown in table 1.

**Table 1:** Morphological and histological characteristics of gonads during different phases of the reproductive cycle in the fish, *M. armatus* (Lacepede).

Reproductive stage	Ovaries	Testes
Phase I or Resting Phase (December–February)	Small, shrunken with reduced vascular supply, finger like projections from the wall called ovigerous folds containing small oocytes and oogonia are present.	Small, almost thread with reduced vascular supply.
Phase II or Preparatory Phase (March–May)	Pale yellowish in colour, small yolky oocyte are visible with nucleoli arranged along the inner surface of the nuclear membrane, Yolk nucleus of Balbiani appears in ooplasm.	Whitish, translucent uneven in size with one end broader. Slight increase in volume and vascular supply.
Phase III or Pre spawning Phase (June–Early July)	Yellow in colour occupying greater space in the abodominal cavity, oocytes clearly visible with distinct nucleus, whole of the ooplasm is filled with protein yolk bodies	Appear turgid and pink in colour due to increased blood supply.
Phase IV or Spawning Phase (Late July to September)	Occupies most of the portion of abdominal cavity, ripe ova are filled with large yolk globules, under abdominal pressure milt oozes.	Testes appears slightly reddish due to maximum increase in blood supply, considerable increase in volume of testis was noted. Due to increased abdominal pressure milt oozes
Phase V or Post spawning Phase (October–November)	Ovaries shrunken in size, collapsed, blood supply reduced, corpora atretica can be seen.	Becomes small in size and translucent, blood supply decreases.

### Plasma Calcium, Sex Steroid and Stanniocalcin Level

#### In males

**Figure 1:**
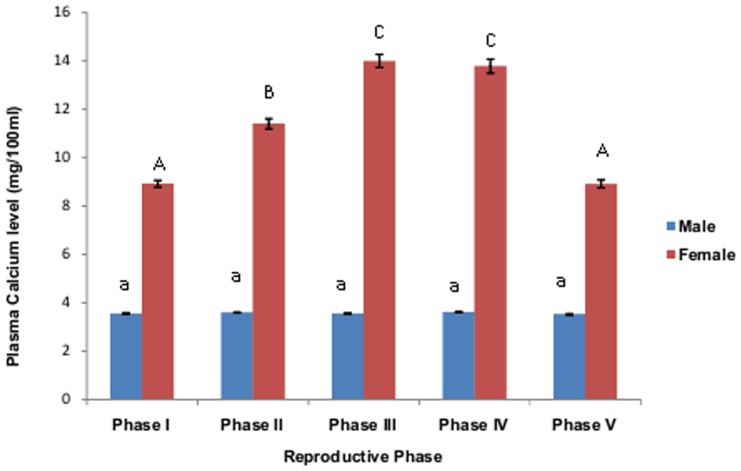
Plasma calciumlevelof male and female *M. armatus* during different phases of gonadal cycle. Each value is mean±SD. Means with different letters are significantly different (P≤0.05).

**Figure 2:**
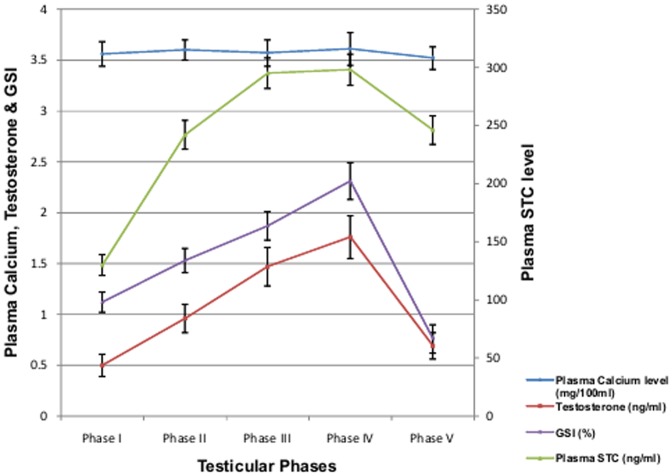
Plasma calcium, stanniocalcin, testosterone level and gonadosomatic index of male *M. armatus* during different phases of testicular cycle. Each value is mean ±SD.

The plasma calcium level estimated during different phases of testicular cycle in the fish *M. armatus* indicates that there exists no correlation between plasma calcium level and testes growth. Negligible variation in plasma calcium level was observed in male fishes as compared to female fishes during various phases of reproductive cycle (Fig. 1). However a considerable variation in plasma testosterone as well as STC level was noted during various reproductive phases in male fishes (Fig. 2). A weak correlation between plasma STC and calcium level (R = 0.3257, y = 621.65x-1978.34), whereas a moderate positive correlation (R = 0.6496, y = 3.52+0.04x) was found between plasma testosterone and calcium level during various phases of testicular cycle. The gonadosomatic index increases from preparatory phase to pre-spawning phase and further decreases after spawning in the post spawning phase which indicates that the gonads undergo increase is weight during prespawning phase and depletion after spawning in the post spawning phase. Testosterone ranged from 0.5–1.7 ng/ml.

#### In females

**Figure 3:**
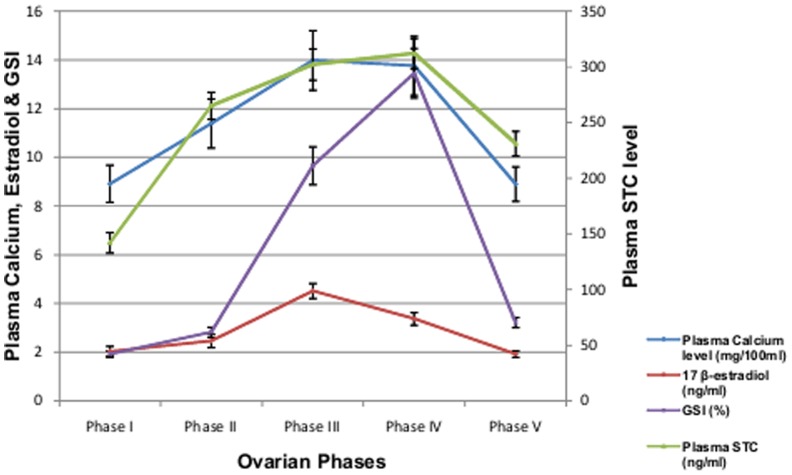
Plasma level of calcium, STC, 17 β-estradiol and Gonadosomatic index of female *M. armatus* during different phases of ovarian cycle. Each value is mean ±SD.

There exists a strong positive correlation between plasma calcium and STC level (R = 0.8763, y = 24.18x-25.01) as well as between plasma calcium and 17 β-estradiol (R = 0.9161, y = 5.39+2.09x) in female *M. armatus* during different phases of reproductive cycle (Fig. 3). Increase in the plasma calcium level was observed during preparatory phase reaching the peak during pre spawning and spawning phase. Afterwards gradually decreases with spawning and reduced to minimum at resting phase. 17 β-estradiol changes along with GSI during ovarian cycle and ranged from 2–4.5 ng/ml. A sudden increase in gonadosomatic index was observed after preparatory phase and reaches maximum during spawning phase after which it decreases.

### Nuclear Diameter of CS Cells

**Figure 4:**
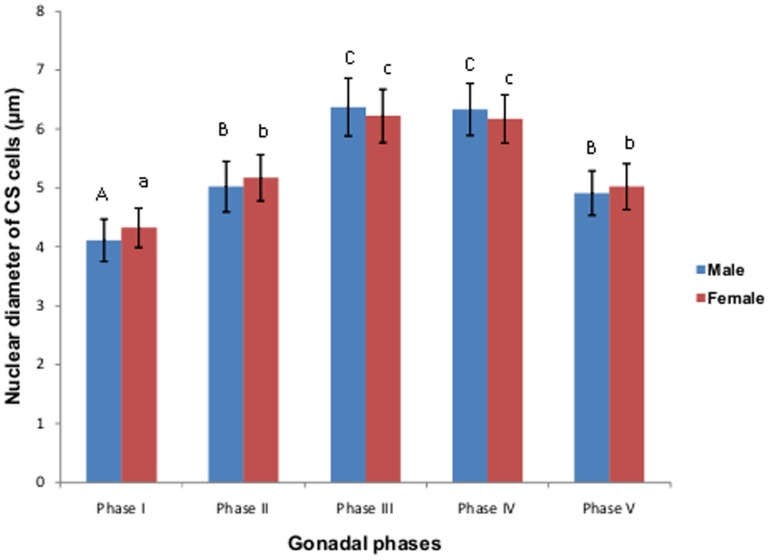
Changes in nuclear diameter of CS cells during different phases of gonadal cycle in male and female*M. armatus*. Each value is mean ±SD. Means with different letters are significantly different (P≤0.05).

Nuclear diameter of CS cells also showed a large variation (Fig. 4). Maximum hypertrophy in nuclei of CS cells was observed during pre-spawning and spawning phase in both male and female fish.

### Effects of Synthetic Steroid Administration

**Figure 5:**
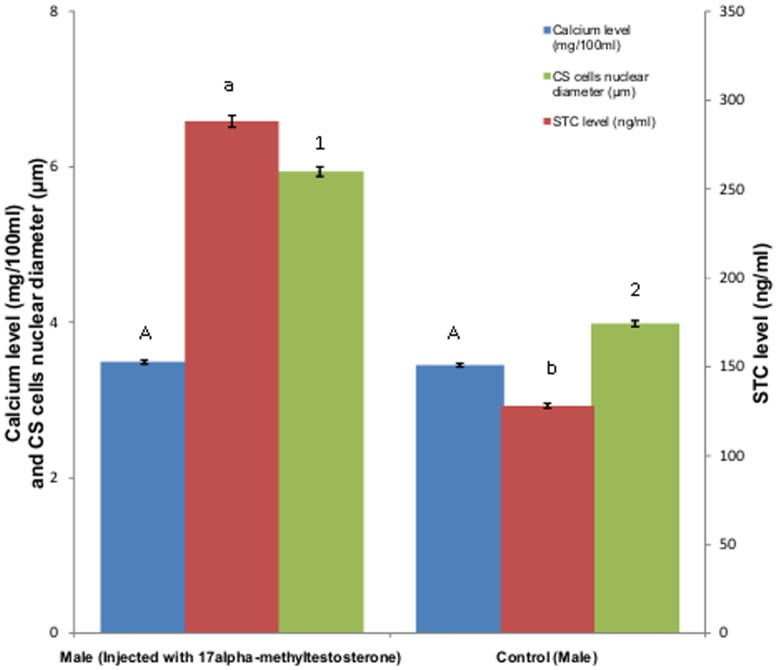
Effect of synthetic steroid administration on plasma calcium and STC level and nuclear diameter of CS cells of male *M. armatus*. Each value is mean ±SD. Means with different letters/number are significantly different (P≤0.05).

**Figure 6:**
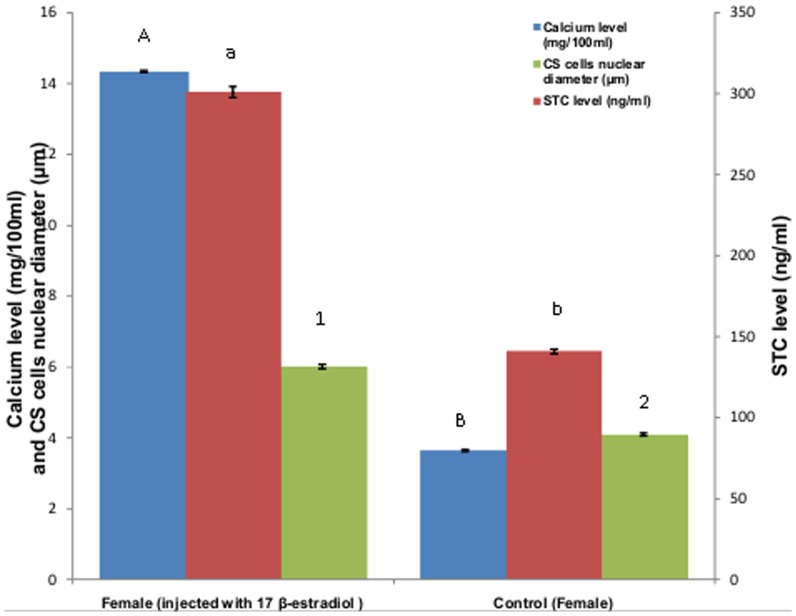
Effect of synthetic steroid administration on plasma calcium and STC level and nuclear diameter of CS cells of female *M. armatus*. Each value is mean ±SD. Means with different letters/number are significantly different (P≤0.05).

The result of synthetic steroid administration in both male and female is shown in Fig. 5 and Fig. 6 respectively. Administration of 17alpha-Methyltestosterone in males resulted in negligible increase in the plasma calcium levels whereas considerable increase in plasma STC as well as nuclear diameter of CS cells was observed. However administration of 17 β-estradiol in females resulted in an increase in plasma calcium level, nuclear diameter of CS cells and plasma STC levels.

## Discussion

In fishes, corpuscles of Stannius synthesizes and secretes stanniocalcin, a hormone involved in calcium homeostasis [4]–[6], but difference in its activity between the sexes during reproductive cycle has been reported in fishes like *O. mossembicus* [27], *Mugil cephalus* [38] and *Notopterus notopterus* [39].

The annual sex cycle of the fish *M. armatus* has been divided into 5 phases on the basis of the variation in the gonadosomatic index and histological features displayed by the testes and ovaries. Variations in the plasma level of 17- β-estradiol in females and testosterone in males during various reproductive phases were observed which showed similar pattern in changes as that of GSI and thus helpful in accessing the seasonal activity of gonads. High values of 17- β-estradiol between late July to September clearly indicates that females would likely to spawn during these months as increased level of 17- β-estradiol initiates vitellogenesis [40]. High levels of testosterone during preparatory phase and spawning phase signified that fishes undergo spermatogenesis and spermiogenesis while low value indicates spermatogonia proliferation [41]. High level of testosterone from August to September in *M. armatus* indicates it produce milts during this period. December to February is the months having low levels of testosterone indicating spermatogonia proliferation phase for *M. armatus*. It has been observed that the seasonal changes in the activity of CS, indicated by increase in the nuclear diameter of CS cells as well as plasma STC level was found to increases in parallel with the growth of ovaries and testis similar to the result obtained previously by Subhedar and Rao in *Heteropreustes fossilis* [42]. There is an increase in the nuclear diameter of CS cells at the beginning of preparatory phase (March). Maximum hypertrophy in nuclei of CS cells was observed during pre-spawning (June- early July) and spawning phase (Late July– September) in both male and female fish. These concomitant changes occurring in the nuclear diameter of CS cells and the gonads definitely suggest a correlation between gonadal maturation and CS activity. A remarkable increase in STC levels were observed in both male and female *M. armatus* during the preparatory phase reaching the peak during pre spawning phase and spawning phase after which it decreases and considerably reduced during resting phase, suggesting that secretory activity of CS is related to gonadal maturation.

Thus a correlative changes between CS cells and gonadal development has been observed in the fish *M. armatus* in the present investigation.

A marked seasonal variation in plasma calcium level was observed in female *M. armatus* associated with ovarian maturation, similar to the result obtained by earlier workers in other fishes [18], [43]. Increase in its level was observed during the preparatory phase reaching the peak during pre spawning phase and spawning phase and considerably reduced during resting phase. This variation in plasma concentration of calcium showed similar pattern in changes as that of plasma STC level. Female fish develop hypercalcemia during sexual maturation due to increased estrogen secretion by ovary [27]. Level of 17-β estradiol also changes with the ovarian cycle as that of calcium level, which is similar to the observations of earlier workers [44], [45]. Previously it was observed that ovariectomy leads to fall in plasma calcium level as well as activity of CS [46] which can be restored by administration of estradiol [47]. Increased level of estrogen during ovarian maturation initiates vitellogenesis by increasing the rate of transcription and translation of vitellogenin in liver [43] as a result of which protein-bound fraction of plasma calcium level rises [28], [44]. Thus increase in total plasma calcium during advance phase of ovarian maturation is due to the appearance of the calcium containing yolk protein precursor vitellogenin in plasma. As the maturation of ovary advances, increase in plasma calcium level occurs because one atom of calcium is associated with every protein phosphate group in vitellogenin complex [48]. Plasma calcium level was also found to be increased after 17- β-estradiol administration. The CS cells are stimulated when plasma calcium rises during the exposure of fish to increased calcium level (Wendelaar Bonga, *et al.*, 1980). Thus CS hyperactivity during ovarian maturation is due to an increase in serum calcium level which in turn is the effect of increased secretion of estradiol [27]. Thus high state of CS activity during prespawning and spawning phase points to the probable hypercalcemia in female M. armatus. Administration of estradiol induced hypercalcemia in the present investigation which stimulates CS to secrete its anti hypercalcemic hormone, STC.

Plenty of research papers concluded the existence of no correlation between plasma calcium level and testicular maturation [26], [39]. However Woodhead [25] was of different opinion who reported a positive correlation between blood calcium level and testicular maturation in arctic cod and sea cod. Our study also indicates plasma calcium almost remains constant throughout and gonadal cycle in male *M. armatus* but plasma STC level changes with testicular cycle. No considerable variations in plasma calcium level along with testicular cycle were observed in male *M. armatus* which simply indicates that STC might be involved in some function other than calcium homeostasis. Administration of 17alpha-Methyltestosterone in males during resting phase resulted in no significant increase in plasma calcium level while plasma level of STC increases. This also supports the fact that STC is not involved in calcium homeostasis rather it may involved in gonadal maturation.

We came to conclusion that STC is involved in testicular maturation in male *M. armatus* and does not play any role in calcium homeostasis while in females it is involved in gonadal maturation as well as calcium homeostasis.
